# The bacterial patterns suggesting the dynamic features of tick-associated microorganisms in hard ticks

**DOI:** 10.1186/s12866-024-03323-3

**Published:** 2024-05-24

**Authors:** Bin Xu, Mengjie Gu, Qunfeng Wu, Chang Shu, Wenbo Tan, Suwen Wang, Zhengwei Zhong, Xiaoling Wang, Jian Li, Jingwen Wang, Yuanzhi Wang, Wei Hu

**Affiliations:** 1 National Key Laboratory of Intelligent Tracking and Forecasting for Infectious Diseases, National Institute of Parasitic Diseases, Chinese Center for Disease Control and Prevention, Chinese Center for Tropical Diseases Research, Shanghai, China; 2 National Institute of Parasitic Diseases, Chinese Center for Disease Control and Prevention (Chinese Center for Tropical Diseases Research), National Health Commission Key Laboratory of Parasite and Vector Biology, WHO Collaborating Center for Tropical Diseases, National Center for International Research on Tropical Diseases, Shanghai, China; 3 Key Laboratory for Prevention and Control of Emerging Infectious Diseases and Public Health Security of the XPCC, School of Medicine, Shihezi University, Shihezi City, Xinjiang Uygur Autonomous Region China; 4 State Key Laboratory of Genetic Engineering, Ministry of Education Key Laboratory of Contemporary Anthropology, Department of Microbiology and Microbial Engineering, School of Life Sciences, Fudan University, Shanghai, China; 5 The institutes of Biomedical Sciences, College of Life Sciences, Inner Mongolia University, Inner Mongolia, Hohhot, China; 6 Hainan Medical University, Haikou, China; 7 Basic Medical College, Guangxi University of Chinese Medicine, Guangxi, Nanning China

**Keywords:** Ticks, Bacterial component, 16S rRNA, Environment, Engorged status

## Abstract

**Background:**

Ticks are blood-feeding significant arthropods that can harbour various microorganisms, including pathogens that pose health risks to humans and animals. Tick-symbiont microorganisms are believed to influence tick development, but the intricate interactions between these microbes and the relationships between different tick-borne microorganisms remain largely unexplored.

**Results:**

Based on 111 tick pool samples presenting questing and engorged statuses including 752 questing tick and 1083 engorged tick from cattle and goats, which were collected in two types of geographic landscape (semi-desert and alpine meadow). We observed significant variations in the composition of tick-borne microorganisms across different environments and blood-engorgement statuses, with a pronounced divergence in symbionts compared to environmental bacteria. Metabolic predictions revealed over 90 differential pathways for tick-borne microorganisms in distinct environments and more than 80 metabolic variations in response to varying blood engorgement statuses. Interestingly, nine pathways were identified, particularly related to chorismate synthesis and carbohydrate metabolism. Moreover, microbial network relationships within tick-borne microorganism groups were highly distinct across different environments and blood-engorgement statuses. The microbial network relationships of symbionts involve some pathogenic and environmental microorganisms. Regression modelling highlighted positive correlations between the *Coxiella* symbiont and related pathogens, while some environmental bacteria showed strong negative correlations with *Coxiella* abundance. We also identified commensal bacteria/pathogens in bacterial cooccurrence patterns. Furthermore, we tested pathogenic microorganisms of each tick sample analysis revealed that 86.36% (1601/1855) of the tick samples carried one or more pathogenic microorganisms, The total carrier rate of bacterial pathogens was 43.77% ((812/1855). Most blood samples carried at least one pathogenic microorganism. The pathogens carried by the ticks have both genus and species diversity, and *Rickettsia* species are the most abundant pathogens among all pathogens.

**Conclusion:**

Our findings underscore that the bacterial pattern of ticks is dynamic and unstable, which is influenced by the environment factors and tick developmental characteristics.

**Supplementary Information:**

The online version contains supplementary material available at 10.1186/s12866-024-03323-3.

## Introduction

Ticks are an important vector that transmit pathogens by biting humans, domestic animals, or wildlife [1, 2]. The compositions of the tick microbiome were reported to be affected by various factors, such as tick species, life stage, hyperaemia level, and habitats and forest structures [3–7]. With the diversity of the microorganisms, there are usually hundreds of thousands of microorganisms in the microbial community carried by ticks [8], which leads to diverse physiological interactions between the tick hosts and their microbes. Some of these bacterial microorganisms have been proven to affect the growth and development of ticks, the blood meal behavior of ticks, and the transmission of pathogens by ticks [9, 10].

Additionally, the relationship between the microorganisms carried by ticks has not been revealed. In nature, distinct relationships, such as coexistence, mutualism, symbiosis, antagonism, competition, parasitism, and predation, exist among different microorganisms [11]. The relationship between microorganisms has been used in human body. For example, with additional intervention, the intestinal microbiota related to metabolism could be changed to achieve the metabolic health of the human host [12, 13]. However, it is a difficult and arduous task to identify the relationship between the microorganisms carried by ticks. While Amplicon sequencing of variable region V3-V4 of 16S rRNA gene technology cannot elucidate complex interaction networks between microorganisms, it can provide insights into macro-level microbial abundance. Utilizing these abundance levels, it is possible to construct an abundance correlation network to unveil trends in microbial abundance changes [14]. Understanding the changes in the abundance of key microorganisms in the tick-borne microbial community, such as symbolic bacteria and tick-borne pathogens (TBP), may help achieve similar intervention to human intestinal, i.e. manually intervene the composition of the tick-borne microbial community. Unfortunate such study is rarely reported.

In this study, We identified and characterized of bacterial microorganisms carried by ticks collected from different locations and different blood-engorgement statuses. Additionally, the bacterial co-occurrence networks of these communities were analyzed to reveal potential interaction patterns of tick-borne bacterial microorganisms.

## Materials and methods

### Sample collection

During the period spanning from late April to mid-May each year in 2019–2020, when adult ticks exhibited their peak activity in the Xinjiang Uygur Autonomous Region (XUAR), northwestern China. These collections encompassed both questing ticks and engorged ticks. The collection sites were situat**e**d in Wenquan County (WQ) (81°08’-81°10’E-44°95’-44°98’N, alpine meadow) and Alataw City (ALSK) (82°48’51’’E-45°04’22’’N, semi-desert). The questing ticks were gathered using the artificial trapping method [15]. The fully engorged ticks were systematically sampled from entire body of each pastured cattle and goat at different intervals, and subsequently preserved in 70% ethanol for further analysis.

With the consent of their owners, we also obtained blood samples from 25 pastured cattle in Alataw City and 79 pastured sheep in WQ County. During the period, we captured 10 great gerbils (*Rhombomys opimus*) from Alataw City and 29 long-tailed ground squirrels (*Spermophilus undulatus*) from WQ County. During the necropsy process, blood samples were collected from each rodent and stored at -80 °C until DNA extraction. Furthermore, we collected 368 blood samples from local herdsmen in WQ County. Detailed information about all the specimens, including their geographical location, host, the number of ticks from animal’s body, and the date of collection, was meticulously recorded (shown in Table 1).

**Table 1:** Information of the samples include ticks and blood sample

Locations	Group	Tick of Status	Species of ticks identified	Number of ticks	Number of tick sample pools (total ticks)	Blood samples	
						Host (*n*)	Human (*n*)
ALSK	ALSKqt	Questing	*Hyalomma asiaticum*	147	15(152)	-	-
			*Dermacentor nuttalli*	4			
			*Hyalomma marginatum*	1			
	ALSKet	Engorged	*Hyalomma asiaticum*	285	40 (571)	livestock cattle (25)	-
			*Hyalomma asiaticum*	230			
			*Hyalomma detritum*	46			
			*Dermacentor nuttalli*	5			
			*Rhipicephalus sanguineus*	2			
			unknow	3			
	-	-	-	-	-	great gerbils (10)	
WQ	WQqt	Questing	*Dermacentor nuttalli*	600	35 (600)		-
	WQet	Engorged	*Dermacentor nuttalli*	512	21(512)	Goats (79)	-
	-	Engorged	*Dermacentor nuttalli*	20	-	long-tailed ground squirrels (25)	-
	-	-	-	-	-	-	368

### DNA extraction and tick molecular species identification

Before DNA extraction, ticks were carefully cleaned by 70% ethanol and phosphate buffered saline (PBS 7.4) to prevent any potential DNA contamination from environmental microorganisms. Then, they were briefly frozen in liquid nitrogen for a minute, after which they were delicately sliced into small pieces using a sterile scalpel. These tick fragments were then meticulously homogenized with a sterile micropestle in 100 µL of lysis solution containing proteinase K. Genomic DNA was extracted from each individual whole tick and blood sample using a DNeasy Blood & Tissue Kit (Qiagen, Shanghai, China) according to the manufacturer’s instructions. The concentration of extracted DNA was quantified with a spectrophotometer (NanoDrop ND-2000; Thermo Scientific).

We amplified fragments of the 16S ribosomal (r) RNA gene (455 bp) and cytochrome oxidase subunit 1 (*CO1*) gene (732 bp) from the DNA of each tick. The primers used for PCR were 16S-F1 (5’-TTAAATTGCTGTRGTATT-3’) and 16S-R1 (5’-CCGGTCTGAACTCASAWC-3) [16] and COX1F (5’-GGAACAATATATTTAATTTTTGG-3’) and COX1R (5’-ATCTATCCCTACTGTAAATATATG-3’) [17]. The PCR product specificity was verified by electrophoresis and Sange sequencing. The sequences were adjusted manually and compared with similar sequences retrieved from the GenBank database using BLASTn (http://www.ncbi.nlm.nih.gov/BLAST/). Phylogenetic relationships were inferred with the maximum likelihood (ML) method in MEGA 7.0 software.

### 16S rRNA fragment sequencing for tick carrying microbiome

Prior to sequencing, each 2–20 tick samples were constructed into a tick pool according to tick species, engorged status, host and their geographical location [20]. A total of 111 tick pools were finally constructed in this study (details in Table 1). Preparation of extracted DNA for pyrosequencing followed the protocols described by Fierer et al., 2008 [18] and Bates et al., 2010 [19]. In brief, a region of the 16S rRNA gene was amplified with the 341F (5’-CCTAYGGGRBGCASCAG-3’) and 806R (5’-GGACTACNNGGGTATCTAAT-3’) primers. Triplicate PCR products were pooled together and normalized in equimolar amounts and then loaded on an Illumina MiSeq PE250 platform for 2 × 250 bp paired-end sequencing.

Raw sequence data generated from 16S rRNA sequencing were further processed. Sequences were trimmed to delete adapters using cutadapt v3.4 (https://cutadapt.readthedocs.io/) and low-quality sequence with vsearch v2.17.1 (https://github.com/torognes/vsearch). which discards sequences whose expected errors per base is greater than 1%. Trimmed sequences were classified with Kraken2 (the second version of the Kraken taxonomic sequence classification system) [20] against the Silva database ver. 138.1 [21]. The abundance of each genus is summarized in Bracken [22].

### Diversity analysis

The microbiome diversity in 4 groups: ALSKqt (questing ticks from Alataw City), ALSKet (engorged ticks from Alataw City), WQqt (questing ticks from Wenquan County) and WQet (engorged ticks from Wenquan County), were analyzed with MicrobiomeAnalyst (https://www.microbiomeanalyst.ca/), an integrated platform to comprehend microbiome data. Alpha diversity was estimated with the Shannon index, which accounts for both richness and evenness [23], and beta diversity was assessed using Bray-Curtis distance metrics and visualized using NMDS (nonmetric multidimensional scaling) to detect differences between groups. Significant differences in alpha and beta diversity among groups were tested by ANOVA (analysis of variance) and ANOSIM (analysis of similarities), respectively. LEfSe (linear discriminant analysis effect size) was then applied to distinguish genera that had different abundances among groups.

### Function prediction of microbial communities

Trimmed sequences in the sequence processing section were clustered into amplicon sequence variants (ASVs) with the UNOISE 3 algorithm [24]. A 97% identity threshold, which corresponds approximately to the taxonomic level of species for bacteria [25], was used to generate ASVs with abundances for functional analysis. The ASV abundance table was delivered to Picrust2 [26] that implements a full pipeline to predict functional abundances of microbial communities based only on marker gene sequences. Pathway abundances, generated through a predictive approach in Picrust2 relying on MinPath, were selected for differential analysis between various groups. To initiate the analysis, the ALSKet group was subdivided into two subsets that had the maximum disparity in the number of each tick species (weighted by proportion). This approach aimed to simulate different tick compositions, allowing us to uncover whether ticks with varying species compositions exhibit distinct microbial community functions. Next, we conducted comparisons between the ALSK and WQ, ALSKqt and WQqt, and ALSKet and WQet groups to investigate the environmental impact on these communities. This analysis helped us to uncover variations in microbiome functions between ticks in these two states. All comparisons were carried out with the statistical analysis software STAMP v2.1.3 [27] using two-sided Welch’s t test and multiple test correction following the Bonferroni method to reduce false positive findings.

### Network inference

The microbial co-occurrence networks (CoNets) help to deduce potential associations among specific genera within ticks. The genus abundance table produced by Bracken was employed to perform pairwise correlation calculations of microorganisms using the Sparse Correlations for Compositional data (SparCC) method, utilizing its default parameters [28]. The threshold value for generating regional CoNets was determined with the random matrix theory method [29], which are 0.56, 0.608, 0.308, 0.332 for group ALSKqt, ALSKet, WQqt and WQet respectively. Next, nodes and edges were generated from the adjacency matrix of pairwise correlations with the R package igraph v1.2.6 [30] in R 4.1.1 [31]. Visualization of networks and analysis of features were performed with Gephi v0.9.2 [32].

### Fitting of the multiple linear regression model

Multiple linear regression was applied for two dominant symbionts, *Coxiella* and *Francisella*, to interpret the abundance change of symbionts with the abundance of their correlated genera. First, correlated genera were obtained from the SparCC correlation matrix for each symbiont, which were both positively and negatively correlated with the symbiont. Next, abundance data of symbionts and their correlated genera were split to obtain a training dataset and a test dataset at a ratio of 7:3. Then, a multiple linear regression model of abundance between the symbiont and its correlated genera was fitted with the function lm in the stats package of R, followed by optimizing the independent variable with a function step to exclude genera that contribute to a limited explanation for the abundance change of the symbiont. The optimized model was finally evaluated with the function gvlma in the gvlma package [33]. The test dataset was used to check the accuracy of the model with the function predict_lm.

### Pathogen examination

All collected samples were tested nine genera of tick-borne pathogens, including *Anaplasma*, *Babesia*, *Bartonella*, *Borrelia*, *Coxiella*, *Ehrlichia*, *Francisella*, *Rickettsia* and *Wolbachia* by conventional PCR. The sequences of the primers used for detecting these pathogens were adopted from previous reports [34–42] and are listed in Additional file 1, while the primers for detecting *Francisella tularensis* (rpoB Fran-F/Fran-R) and *Ehrichia spp.* (*Ehrlichia* 16S rRNA EBm52F/EBm52R) were self-designed. The optimization steps of the PCR program were adapted from literature as follows: (1) Different concentrations of plasmids carrying the target gene were used as templates to optimize the reaction conditions and test the sensitivity of the primers. (2) Based on the first step, plasmids with the concentration that was the most sensitive to the reaction were selected and mixed with 60 ng DNA of tetracycline-treated ticks to form a template for mimicking the positive infection samples. Template containing 60 ng of DNA from tetracycline-treated ticks and template containing no DNA were used as control groups. Then, all these templates were used to test the specificity and sensitivity of the primers in complex sample situations.

Through experimental optimization and evaluation of the results, the optimum reaction conditions were established for the 11 pairs of primers. The volume of the reaction system for the PCR test was 30 µL in total, containing 27 µL of 1.1×T3 PCR mix, 1 µL of forward primer, 1 µL of reverse primer, and 1 µL of DNA template. The reaction program was set as follows: 1 cycle at 98 °C for 3 min, 35 cycles of 98 °C for 10 s, 54–60 °C for 10 s, 72 °C for 10 ∼ 25 s, and 1 cycle at 72 °C for 2 min. All samples were tested with all primers. The samples that tested positive for *Francisella spp*. and *Ehrlichia spp*. Ehr 521/Ehr 747 were further tested with the primers *Francisella tularensis*_Fran-F/Fran-R and* Ehrlichia spp*. EBm52F/EBm52R, respectively. Samples that tested positive with any primers were subjected to Sanger sequencing to identify their species. In addition, the species of the *Francisella tularensis*-positive samples were further identified by qPCR [43].

## Results

### Sampling and tick species identification

A total of 1855 ticks were collected, including questing ticks (*n* = 752) and engorged ticks (n = 1083) from livestock cattle and goats. 20 ticks (WQrt) were collected from the long-tailed ground squirrels (Table 1). EDTA-anticoagulated blood specimens were collected from parasitized livestock cattle (ALSKcb, *n* = 25) and goats (WQgb, *n* = 79). We also collected blood samples from great gerbils (ALSKrb *n* = 10) and long-tailed ground squirrels (WQrb *n* = 29), and 368 (WQhb) local herdsman blood samples were collected.

Among the 1855 ticks examined, we identified three genera and six species. Notably, 1132 ticks, comprising both questing ticks (*n* = 600) and engorged ticks from livestock goats (*n* = 512) and the long-tailed ground squirrels (*n* = 20), were collected in the WQ area. These ticks were conclusively identified as *Dermacentor nuttalli*. In our study, we observed the presence of two or three different tick species on each livestock animal. Among the 571 engorged ticks examined, we found that 49.91% were *Hyalomma asiaticum* (*n* = 285), 40.28% were *Dermacentor marginatus* (*n* = 230), and 8.07% were *Hyalomma detritum* (*n* = 46), while only a small number of ticks belonged to *Dermacentor nuttalli* (*n* = 5) and *Rhipicephalus sanguineus* (*n* = 2). Three ticks could not be identified. Of the 152 questing ticks, 147 were identified as *Hyalomma asiaticum*, 4 were identified as *Dermacentor nuttalli*, and 1 was identified as *Hyalomma marginatum* (*n* = 1) (for details see Table 1).

### 16S partial region sequencing

Out of the 111 tick pools comprising a total of 1855 ticks, we obtained a total of 4,770,842 quality-filtered reads. On average, each sample yielded approximately 42,981 reads, with a standard deviation of 22,548 and a range spanning from 19,265 to 65,352. The quality of these reads, indicated by an average quality score of 36, was deemed satisfactory. It is noteworthy that all libraries generated from tick samples exhibited ample sequencing depth for subsequent analysis. This is evident from the rarefaction curves plotting the number of observed OTUs, which reached a plateau when considering a sequencing depth ranging from 5000 to 10,000 sequences (Additional file 2). This plateau suggests that our samples had achieved sufficient coverage, validating their suitability for further analysis.

### Microbiome profile

At the phylum level, tick pools in Alataw City carried more diverse bacteria. that those in Wenquan County The shared bacteria between ticks from the two locations belong to 9 phyla: Acidobacteriota, Actinobacteriota, Bacteroidota, Deinococcota, Firmicutes, Gemmatimonadota, Proteobacteria, Fusobacteriota and Verrucomicrobiota, and there were 11 exclusive phyla in ALSK ticks but only one exclusive phylum in WQ ticks (Additional file 3). Regarding abundance differences among groups, ticks from the two environments showed bacterial differences in 14 phyla out of all 21 phyla (Additional file 4). Specifically, questing ticks from ALSK carried more Acidobacteriota (W = 325, *P* = 0.0117), Actinobacteriota (W = 447, *P* < 0.0001), Bacteroidota (W = 426.5, *P* = 0.0005), Cyanobacteria (W = 297.5, *P* = 0.0315), Gemmatimonadota (W = 297.5, *P* = 0.0315) and Patescibacteria (W = 420, *P* < 0.0001) than WQ ticks. In addition, engorged ones from ALSK carried more Actinobacteriota (W = 807.5, *P* < 0.0001), Bacteroidota (W = 775, *P* < 0.0001), Fusobacteriota (W = 516, *P* = 0.0367) and Patescibacteria (W = 577.5, *P* = 0.0016) but less Deinococcota (W = 188, *P* = 0.0004) and Proteobacteria (W = 269, *P* = 0.0215). Regarding questing ticks and engorged ticks, engorged tick pools carried more diverse bacteria than questing tick pools. There were 13 shared bacterial phyla and no exclusive phyla in questing ticks but 8 exclusive phyla in engorged ticks (Additional file 5). Furthermore, questing ticks from ALSK carried more Acidobacteriota (W = 363, *P* = 0.0291) and Deinococcota (W = 493, *P* = 0.0003) but less Fusobacteriota (W = 217.5, *P* = 0.0265) and Verrucomicrobiota (W = 232.5, *P* = 0.0494) than engorged ticks. The questing ticks from WQ carried more Deinococcota (W = 525, *P* = 0.0071) but less Bacteroidota (W = 232.5, *P* = 0.0059) and Firmicutes (W = 209, *P* = 0.0067) than engorged tick pools.

**Fig. 1:**
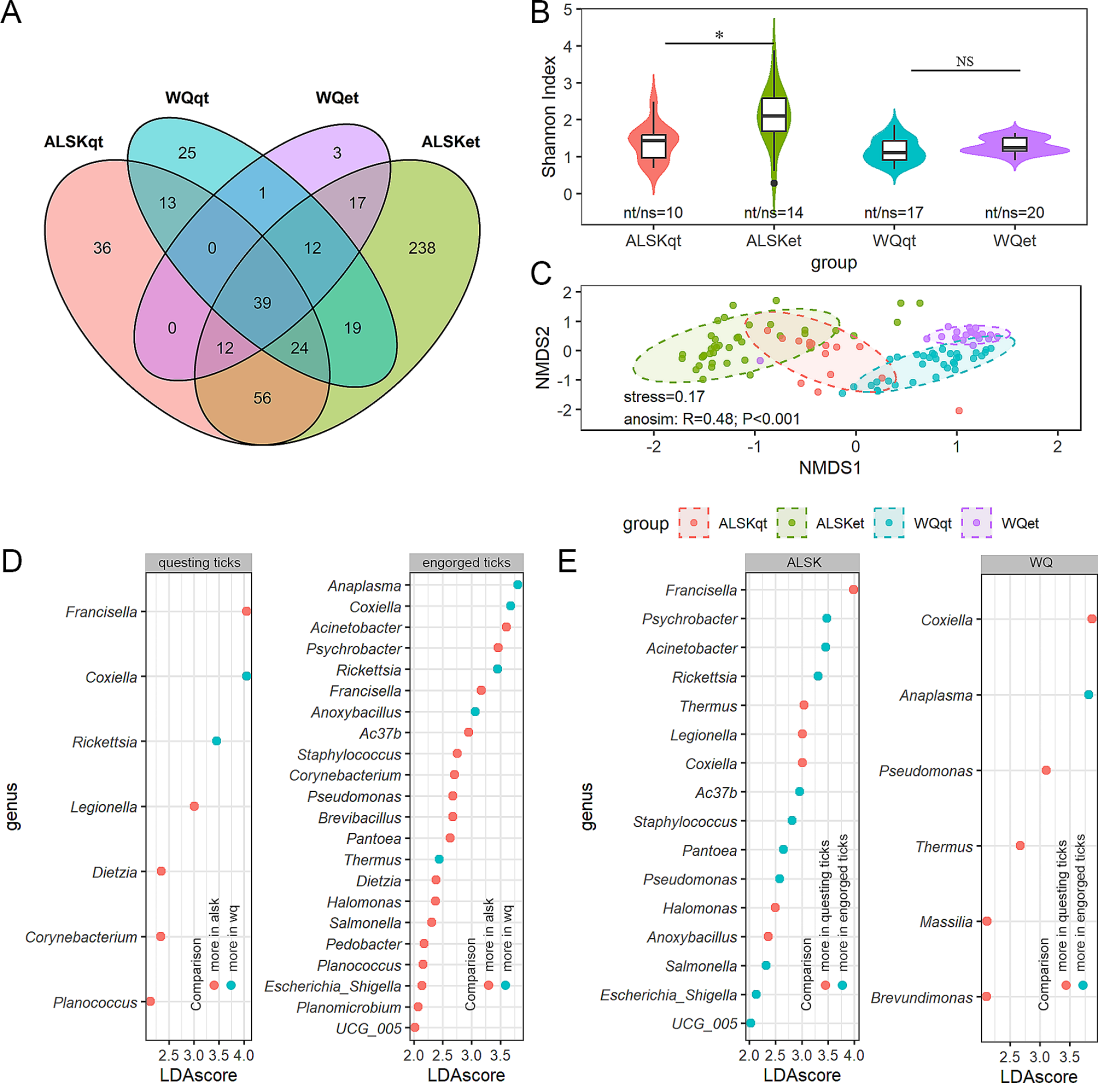
Profiles of the bacterial microbiome from ticks at the genus level. (**A**) Venn diagram of bacterial genera for 4 groups of ticks: questing ticks from ALSK (ALSKqt), engorged ticks from ALSK (ALSKet), questing ticks from WQ (WQqt) and engorged ticks from WQ (WQet); (**B**) Alpha diversity of bacterial genera for the 4 groups of ticks above; (**C**) Beta diversity of bacterial genera for the 4 groups of ticks above; (**D**) Bacterial genera with different abundances between environments; (**E**) Bacterial genera with different abundances between engorgement statuses

At the genus level, the abundance table consisted of 495 genera. Similar to the phylum level, tick pools in Alataw City carried more diverse bacteria than those in WQ County, and engorged tick pools carried more diverse bacteria than questing tick pools (Additional file 6–7). For the 4 groups, there were 203.5 ± 147.63 (mean ± standard deviation) genera for each. Thirty-nine (7.88%) were shared in all groups (Fig. 1A). The shared genera occupied 86.21% of the total abundance. The engorged tick pools in Alataw City had the most exclusive genera (238, 78.81% in all exclusive genera), and 17 among these (7.15%) were from exclusive phyla of this group. While the engorged tick pools in WQ County had the least exclusive genera (3, 0.99% in all exclusive genera), one of these belongs to the only exclusive phylum of this group. In general, the genera with the top 10 relative abundances were *Coxiella* (22.99%), *Rickettsia* (17.90%), *Acinetobacter* (12.23%), *Francisella* (9.65%), *Anaplasma* (5.94%), *Psychrobacter* (5.25%), *Thermus* (3.19%), *Pseudomonas* (2.92%), *Anoxybacillus* (2.39%) and *Rickettsiales* bacterium Ac37b (1.74%) in all 111 samples. These high abundance genera are included in 39 shared genera except the genus *Anaplasma*, and 8 in 10 belong to the phylum Proteobacteria, which showed that shared genera formed a major component of the tick-carrying bacterial microbiome. The diversity of the bacteria within individual samples (alpha diversity) shows richer in engorged tick pools in ALSK (*P* = 5.4e-6) (Fig. 1B). However, the alpha diversity of bacteria in WQ ticks displayed no difference between questing and engorged tick pools (*P* = 0.37). The beta diversity between groups was assessed using the Bray–Curtis distance metrics and visualized using NMDS (Fig. 1C). In beta diversity observations, the genera of the 4 groups were significantly distinct from each other (ANOSIM, *p* < 0.01). LEfSe determined genera that had different abundances between groups (Fig. 1D-E). It showed 7 differential genera between questing tick-carrying bacteria from two places and 22 between engorged tick-carrying bacteria (Fig. 1D). Among these, the top differential genera according to LDA score were intracellular bacteria. However, the most differential genera were environmental bacteria. Two common differential genera that occurred in both places were *Coxiella* and *Thermus*, which are more abundant in questing ticks. For different engorged statuses, there were 16 and 6 differential genera between questing ticks and engorged ticks from ALSK and WQ (Fig. 1E). Similarly, intracellular bacteria were the top differential genera, and the most differential genera were environmental bacteria.

### The predicted function of microbial communities

Four groups were used to detect differences in the functions of microbial communities to inspect the relation of environment and parasite statuses on observed microbial communities. First, 40 samples of the ALSKet group were divided into two subsets, both of which contain 20 samples but have different tick compositions. In the two subsets, one consisted of 0.69% *D. nuttalli*, 61.51% *Hy. asiaticum*, 27.84% *D. marginatum*, 9.62% *Hy. detritum* and 0.34% *R. sanguineus* (*n* = 291), and the other subset consisted of 1.08% *D. nuttalli*, 38.27% *Hy. asiaticum*, 53.79% *D. marginatum*, 6.5% *Hy. detritum* and 0.36% *R. sanguineus* (*n* = 277). Statistical analysis showed no significant change in the functional abundance of microbial communities (Additional file 8), suggesting that tick species have a limited influence on the formation of microbial functions in ticks.

**Fig. 2:**
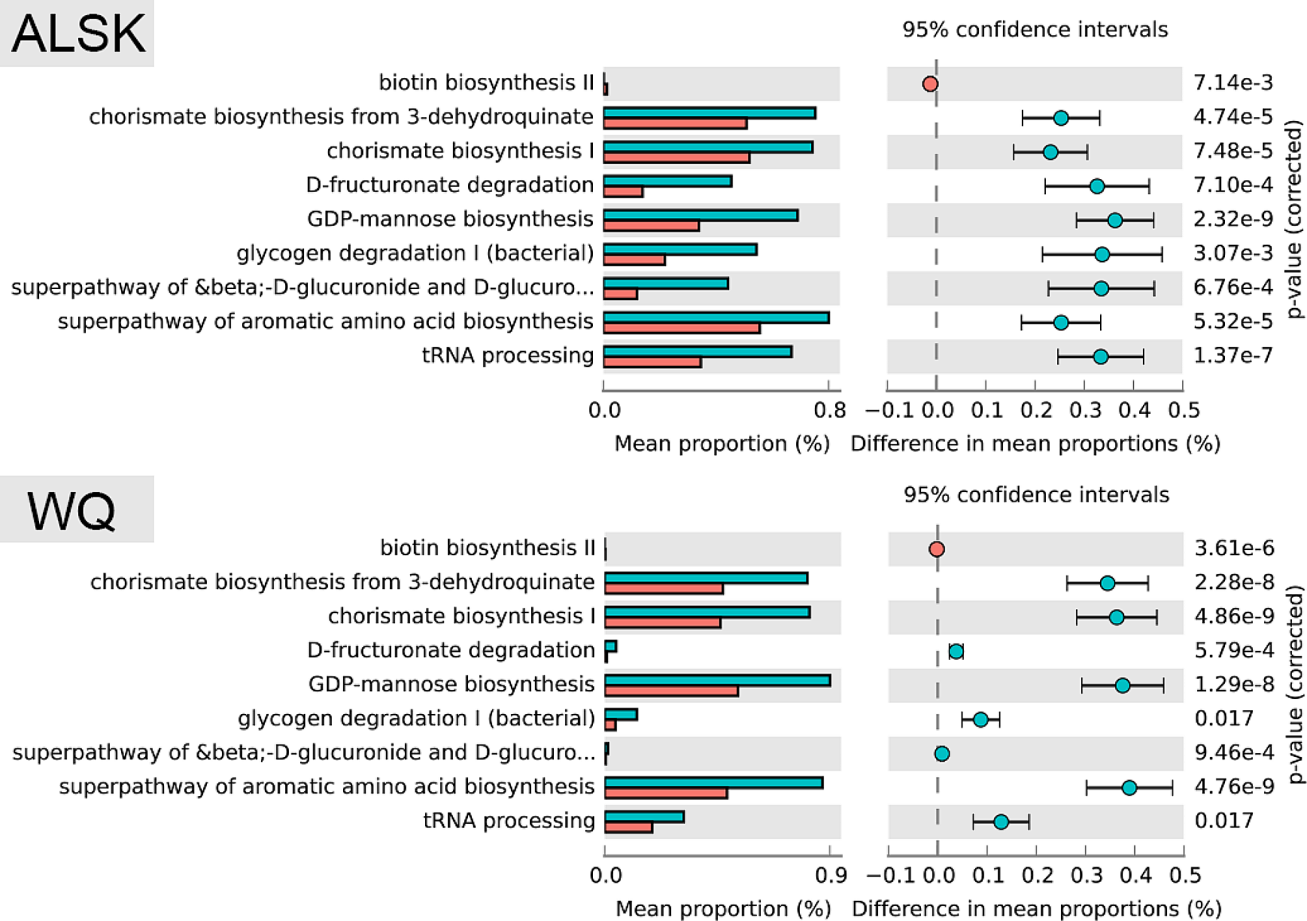
The 9 shared differential metabolic pathways between questing ticks and engorged ticks from two environments. Bar plot with extended error bar. Red and blue objects represent groups of questing ticks and engorged ticks, respectively

For ALSK and WQ tick pools, there were 222 differential function pathways in bacteria (Additional file 9). When engorged statuses were considered, there were 97 detected in questing ticks and 237 detected in engorged ticks (Additional file 10–11). This means that there are many metabolic differences among tick-carrying bacteria in different environments. By inspecting microbial function abundance between ALSKqt and ALSKet, WQqt and WQet, 106 and 83 differential function pathways were discovered (corrected *P*s < 0.05, Additional file 12–13), respectively. Among these pathways, there were 9 shared differential function pathways (Fig. 2), including chorismate biosynthesis I, chorismate biosynthesis from 3-dehydroquinate, the superpathway of β-D-glucuronide and D-glucuronate degradation, glycogen degradation I (bacterial), GDP-mannose biosynthesis, D-fructuronate degradation, the superpathway of aromatic amino acid biosynthesis and tRNA processing, which were all less abundant in engorged ticks, and one pathway of biotin biosynthesis, which was more abundant in engorged ticks.

### Difference between microbial co-occurrence networks (CoNets) of questing and engorged ticks

**Fig. 3:**
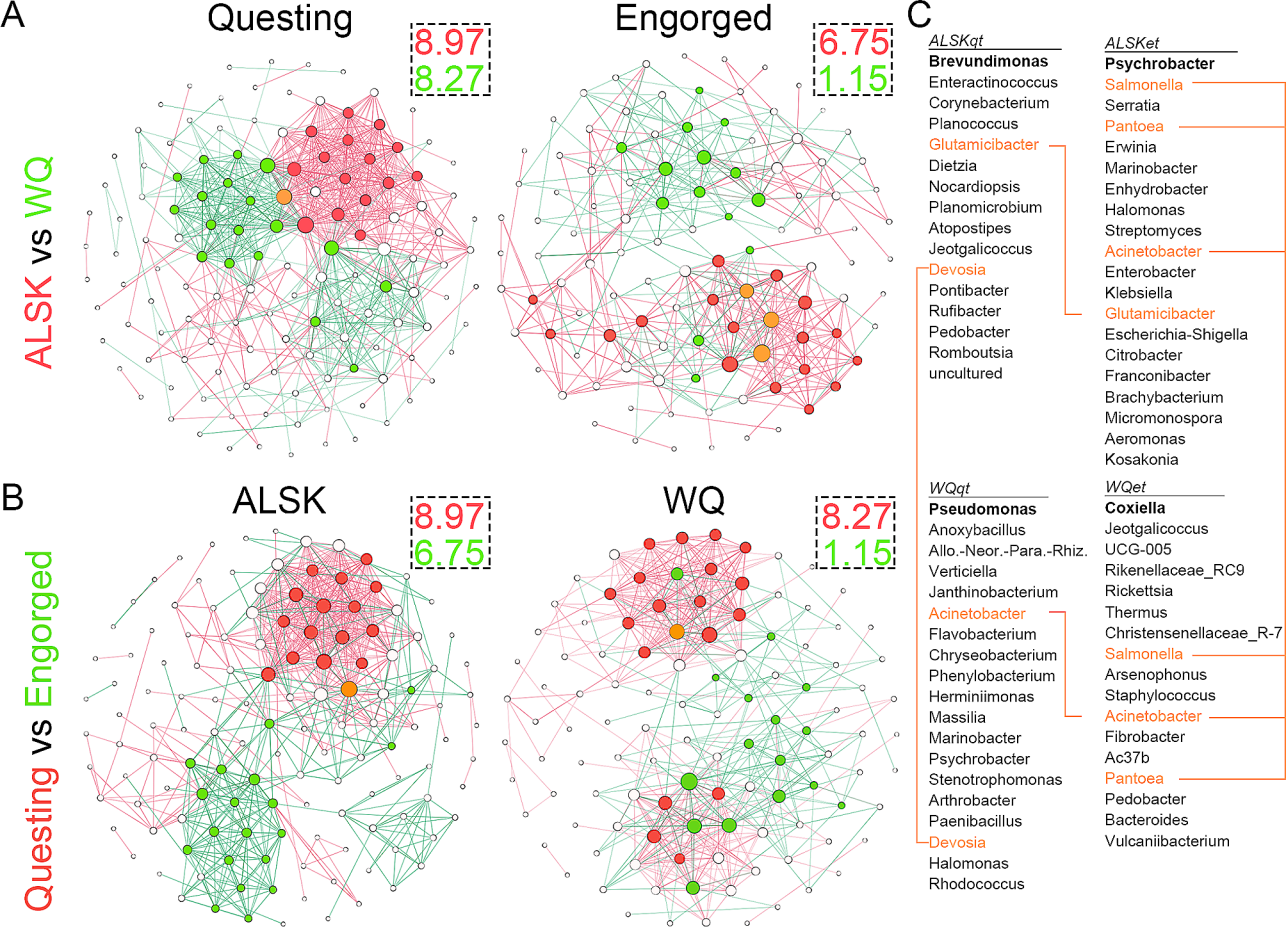
Comparison between microbial co-occurrence networks for tick-carrying bacteria at different levels. (**A**) microbial co-occurrence networks for ticks from ALSK and WQ, left for group questing ticks, right for engorged ticks; (**B**) microbial co-occurrence networks for questing and engorged ticks, left for group ALSK, right for group WQ; (**C**) dominated genera which account for 50% total degree of net of each group, orange lines indicated shared key genera between two groups. Each ball represents a genus, and the size of the ball indicates the degree of this genus. Red and green balls indicate dominant genera of different groups, which account for 50% of the total degree of net. Orange balls indicate shared dominant genera for both groups. Links represent co-occurrence relationships between genera, and their colours show which group displayed this relationship. Values in the dashed box indicate the average degree of the color corresponding group in the cooccurrence network

Microbial co-occurrence networks representing different groups were constructed, and the degree parameters of CoNets are displayed in Fig. 3. The vertices and average degrees of the CoNets of tick-carrying bacteria on questing and engorged ticks of ALSK are less than those of WQ. Similarly, the vertices and average degrees of the CoNets of tick-carrying bacteria on engorged ticks are less than those on questing ticks. These results suggest that the CoNets of tick-carrying bacteria were affected by the environments where ticks grow and in the engorgement status of the ticks.

All groups were composed of different taxonomic profiles and were dominated by the phylum Proteobacteria (Additional file 14). For the abundance of bacteria in CoNets, ticks from two places showed bacterial differences in 5 phyla out of all 10 phyla. Specifically, questing ticks from ALSK carried more Actinobacteriota (W = 417, *P* < 0.0001), Bacteroidota (W = 393, *P* = 0.0004), Firmicutes (W = 329, *P* = 0.0391), and Patescibacteria (W = 391, *P* < 0.0001) and less Proteobacteria (W = 134, *P* = 0.0176) than WQ ticks. In addition, engorged ones from ALSK carried more Actinobacteriota (W = 749.5, *P* < 0.0001), Bacteroidota (W = 709, *P* < 0.0001), and Patescibacteria (W = 540, *P* = 0.0018) and less Deinococcota (W = 172, *P* = 0.0005) and Proteobacteria (W = 112, *P* < 0.0001). Additionally, questing ticks from ALSK carried more Deinococcota (W = 458, *P* = 0.0002) and Proteobacteria (W = 418, *P* = 0.0028) than engorged ticks. Questing ticks from WQ carried more Deinococcota (W = 487, *P* = 0.0079) but less Bacteroidota (W = 463, *P* = 0.0224) and Firmicutes (W = 188, *P* = 0.0059) than engorged ticks. For dominant genera that account for 50% of the total degree of net, few of the same genera were detected between different places or different engorgement statuses. These results showed that the tick-carrying bacteria included in CoNets between the two environments had significant differences in taxonomy and abundance. Meanwhile, in same environment, no pattern for bacterial differences between tick-questing and engorged ticks was detected.

### Pathogen-related genera correlated to symbionts *Coxiella* and *Francisella*

**Fig. 4:**
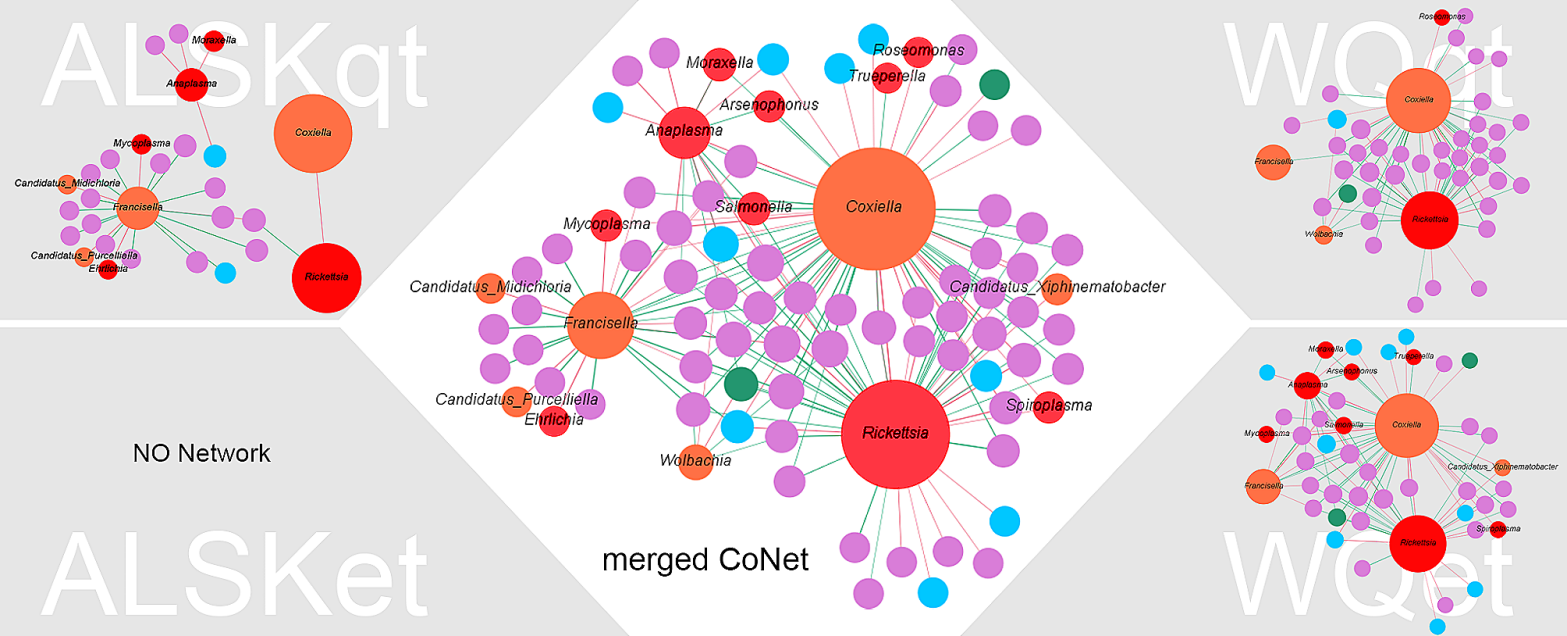
Genera correlated with endosymbionts *Coxiella* and *Francisella*. Each ball represents a genus, and the size of the ball indicates the degree of this genus. Orange, red, purple, blue and green balls represent endosymbionts, pathogen-related genera, environmental bacteria, gut bacteria and unknown genera, respectively. Names of endosymbionts and pathogen-related genera are displayed

After extracting local correlations for pathogen-related genera and 2 high abundance symbionts (*Coxiella* and *Francisella*) from the SparCC correlation matrix, a correlation network was summarized, as shown in Fig. 4. There were 63 connected to *Coxiella* in a total of 85 genera and 28 connected to *Francisella*. Among these, 44 environmental genera, 9 pathogen-related genera, 6 gut-related genera, 2 symbiont genera and 2 undetermined genera were connected to *Coxiella*. In addition, 23 environmental genera, 2 pathogen-related genera, 1 gut-related genus and 2 symbiont genera were connected to *Francisella*. The remaining 21 genera connected to *Coxiella* were mediated by one genus, but *Francisella* still had 10 genera that were not connected to *Coxiella*. Pathogen-related genera that were positively correlated with *Coxiella* were *Anaplasma*, *Mycoplasma*, *Rickettsia*, *Roseomanos*, *Spiroplasma* and *Ehrlichia*. *Arsenophonus*, *Moraxella*, *Samonella*, and *Trueperella* were negatively correlated with *Coxiella.* In addition, *Ehrlichia* and *Mycoplasma were* directly correlated with *Francisella*, both positively, and 5 indirectly correlated with *Coxiella*. These results revealed that the main correlations of symbionts were environmental genera, and some important pathogen-related genera correlated to symbionts where these genera showed different correlations with each symbiont. Additionally, no stable pattern of relationships between the two symbionts exists in different environments or engorged statuses.

### Multiple linear regression model (MLRM) revealed a quantitative relation between *Coxiella* and its correlated genera

**Fig. 5:**
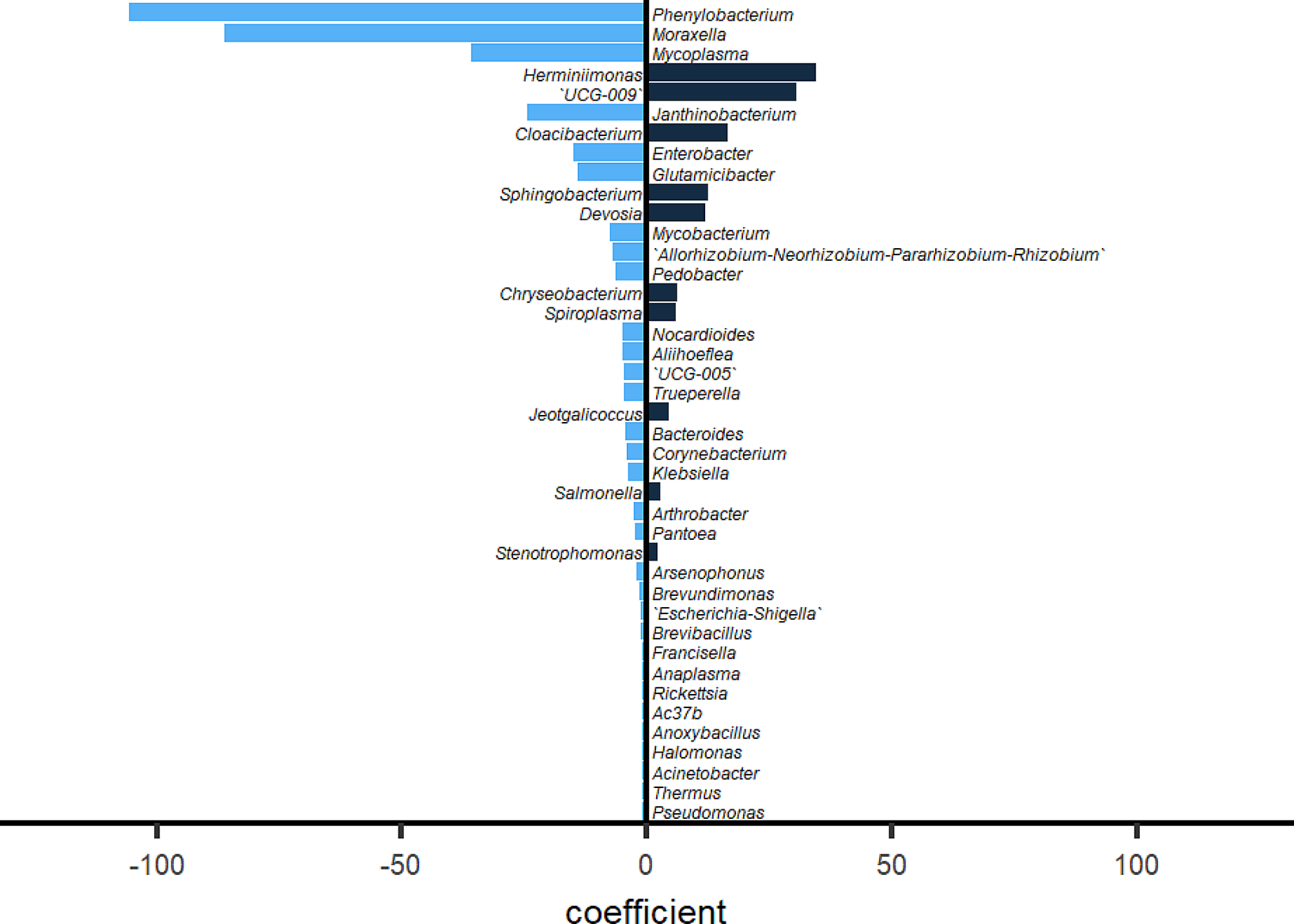
The 41 genera and their coefficients that have abundance correlations with *Coxiella* revealed by a multiple linear regression model. Negative and positive coefficients indicate negative and positive correlations with *Coxiella*, respectively. The absolute value of the coefficient indicates the weight of the correlation with *Coxiella*

For the two symbionts, only the dataset of *Coxiella* passed the evaluation of MLRM in gvlma, which suggested that the abundance change between *Francisella* and its correlated genera did not satisfy a linear relation. The MLRM formula for the abundance of *Coxiella* comprised 7 intracellular parasites, 6 gut-related genera and 28 environmental genera (Fig. 5). The abundance of these 41 genera interpreted a 99.97% change in *Coxiella* abundance. For the test dataset, the R squared between the prediction of the model and observation was 0.7280 after the negative predicted value was corrected to 0. The top 3 coefficients with the most weights, which belong to the genera *Phenylobacterium*, *Moraxellla* and *Mycoplasma*, were all negative. The top 3 positive coefficients with the most weights were possessed by the genera *Herminiimonas*, *UCG-009* and *Cloacibacterium*. The weight proportions of the 6 genera in the formula were 21.96%, 17.92%, 7.43%, 7.18%, 6.34% and 3.46%. These results revealed a quantitative relation between *Coxiella* and its correlated genera, and environmental genera were the most important factors influencing the abundance of symbionts of *Coxiella*.

### Pathogens and symbionts in ticks

A total of 8 microorganisms with different carrier rates were detected in samples from 10 sources, including pathogens and tick symbionts: *Ehrilichia spp*. (0.42%, 10/2336), *Bartonella spp*. (0.77%, 18/2336), *Babesia spp*. (5.77%, 135/2336), *Borrelia spp*. (7.49%, 175/2336), *Francisella spp*. (19.34%, 452/2336), *Anaplasma spp*. (21.48%, 502/2336), *Rickettsia spp*. (30.52%, 713/2336), and *Coxiella spp*. (52.48%, 1226/2336). In addition, 86.36% of the tick samples carried one or more of the detected bacterial microorganisms, and the carrying rates from low to high were 20% (4/20) for WQrt, 75.66% (432/571) for ALSKet from livestock cattle, 87.33% (524/600) for WQqt, 95.70% (490/512) for WQet from goats and 100% (152/152) for ALSKqt. Moreover, 43.77% (812/1855) of tick samples carried bacterial pathogens, and nearly 18% of blood samples carried one or two detected pathogens. More specifically, the bacterial pathogen carrying rates of the blood samples from low to high were 0% (0/29) for the WQrb, 0% (0/10) for the ALSKrb, 1.6% (6/368) for the WQhb, 46% (13/28) for the ALSKcb and 94% (74/79) for the WQgb (for details see Table 2).

**Table 2:** Detection the statistics of individual microbes within each sample type

Target pathogens	Sample size	Matched species	Positive samples	Distribution									
				WQ						ALSK			
				WQqt	WQet	WQgb	WQrt	WQhb	WQrb	ALSKqt	ALSKet	ALSKcb	ALSKrb
*Coxiella* spp.	1226	Uncultured *Coxiella* sp. clone Dx-56	1226	485	393	0	2	0	0	101	245	0	0
*Rickettsia* spp.	713	*Rickettsia spp*.	314	110	127	0	2	0	0	4	70	1	0
		*Rickettsia raoultii*	229	147	39	0	0	0	0	0	43	0	0
		*Rickettsia sibirica*	53	18	25	0	0	0	0	0	10		0
		*Rickettsia* sp. Hme_HirooL009	4	3	0	0	0	0	0	0	1	0	0
		Uncultured *Rickettsia* sp.	111	33	20	0	0	0	0	0	58	0	0
		*Rickettsia* sp. BJ-900	2	0	2	0	0	0	0	0	0		0
*Anaplasma* spp.	502	*Anaplasma ovis*	416	11	323	74	0	0	0	0	8	0	0
		*Anaplasma phagocytophilum*	86	52	19	0	0	0	0	0	15	0	0
*Francisella* spp.	452	*Francisella* endosymbiont	448	71	11	0	0	5	0	146	215	0	0
		*Francisella tularensis* subsp.	4	0	0	0	0	0	0	0	4	0	0
*Borrelia* spp.	175	*Borrelia burgdorferi*	175	15	88	0	0	0	0	8	64	0	0
*Babesia* spp.	135	Uncultured *Babesia* G50 gene for 18 S rRNA, partial sequence	135	55	67	0	0	0	0	0	2	11	0
*Bartonella* spp.	18	*Bartonella henselae* strain Houston-I	18	9	9	0	0	0	0.	0	0	0	0
*Ehrilichia* spp	10	*Ehrlichia* sp. strain WHBMXZ-40	10	1	5	1	0	2	0	0	1	0	0

### *Ehrilichia* spp.

Sanger sequencing analysis showed that these *Ehrilichia* sequences were the same and had 100% homology with 16S rRNA sequence of *Ehrilichia* (JX402605.1) found in *Hy*. *asiaticum* ticks in Xinjiang Province (northwestern China), the *Ehrilichia* 16S rRNA sequence KX987325.1 carried by *Boophilus microplus* ticks in Hubei Province (central China) and the *Ehrilichia* 16S rRNA sequence KY046298.1 carried by *Rhipicephalus microplus* ticks in Malaysian [44]. By aligning the sequences with the Basic Local Alignment Search Tool (BLAST), it was found that this *Ehrilichia* sequence had 100% homology with MT875368.1 (Candidatus *Ehrlichia Hainanensis*), KJ513197.1 (*Ehrlichia canis*), MZ733621.1 (Candidatus *Ehrlichia pampeana*) and MT738235.1 (*Ehrlichia ruminantium*). These results suggest that this species may be a pathogen that is prevalent in humans, livestock, and rodents.

### *Bartonella* spp.

*Bartonella spp*. were detected only in tick samples collected from Wenquan County. The results of Sanger sequencing showed that *Bartonella spp*. had 100% homology with *Bartonella henselae*. *Bartonella henselae* is a zoonotic pathogen causing neurological disease [45]. However, this pathogen was not detected in the human blood and goat blood samples collected from Wenquan County. Since the felines and canines in Wenquan County were not included in the scope of this investigation, the situation was still unknown.

### *Babesia* spp.

*Babesia* spp. was detected in a total of 136 cases in ticks, livestock and human blood samples from Wenquan County and Alataw City. By sequencing and sequence alignment, it was found that the sequence of the *Babesia* spp. in questing ticks and parasitic ticks had 100% homology with LC553515.1, which is a *Babesia* strain closely related to the zoonotic *Babesia* gibsoni [46].

### *Borrelia* spp.

*Borrelia spp.* were detected in a total of 175 ticks. The results of Sanger sequencing showed that all *Borrelia spp.* had the same sequence, which was 100% homology with the NR_170496.1 sequence of *Borrelia maritima*. *B. maritima* was a new species of the *Borrelia burgdorferi sensu lato* complex [47, 48].

### *Francisella* spp.

*Francisella spp.* was detected in a total of 452 samples (5 cases in human blood samples and others in tick samples). The results of Sanger sequencing showed that 4 cases of Alataw City engorged ticks from livestock cattle carried *Francisella tularensis subsp.* (CP009653.1), and the others carried *Francisella*-like symbionts (KX852466), which is a tick symbiont that is transmitted vertically through the maternal line. These results suggest that *Francisella*-like symbionts are stable in the population of ticks in Alataw City.

### *Anaplasma* spp.

*Anaplasma spp.* was detected in a total of 502 samples. Two species were identified by Sanger sequencing and sequence alignment, including *Anaplasma ovis* (NZ_CP015994.1), which was found in 416 cases, and *Anaplasma phagocytophilum* (NC_021880.1), which was found in 86 cases. *Anaplasma ovis* is the *Anaplasma* (Rickettsiales: Anaplasmataceae) of susceptible goat. The positive rate of goat blood samples collected from Wenquan County was 93.67% (74/79), and the positive rate of engorged ticks from goat, was 63.08% (323/512), suggesting that the goats that are raised in Wenquan County should be further tested to prevent the spread of pathogens. Moreover, *Anaplasma phagocytophilum*, an intracellular parasite that can cause human granulocytic anaplasmosis, was detected in tick samples from both places [49], suggesting that the prevention and control of *A*. *phagocytophilum* infection in both Wenquan County and Alataw City need to be reinforced.

### *Rickettsia* spp.

*Rickettsia spp.* was detected in a total of 713 samples. Among those cases, only 1 case was from cattle blood samples in Alataw City, and the rest were from tick samples. Six species were identified by next-generation sequencing, and the sequence alignment results indicated that 5 of the six species were zoonotic pathogens, including *Rickettsia raoultii (*229/713) [50], *Rickettsia sibirica* (53/713) [50], *Rickettsia sp.* Hme_HirooL009 (4/713) (LC544134.1), uncultured *Rickettsia sp.* (KM587631.1; KM587632.1; KM587633.1; MT434895.1; KX591658.1) (111/713) [51] and *Rickettsia sp*. BJ-90 (2/713) [52]. *Rickettsia sp.* BJ-90 is a pathogen identified as belonging to the *Rickettsia sibirica* clade by evolutionary analysis. Taken together, the carrying rate of the *Rickettsia* pathogen in ticks was 55.96%. The *Rickettsia* species of the remaining 314 samples could not be determined, accounting for 44% of the total sequences.

### *Coxiella* spp.

**Fig. 6:**
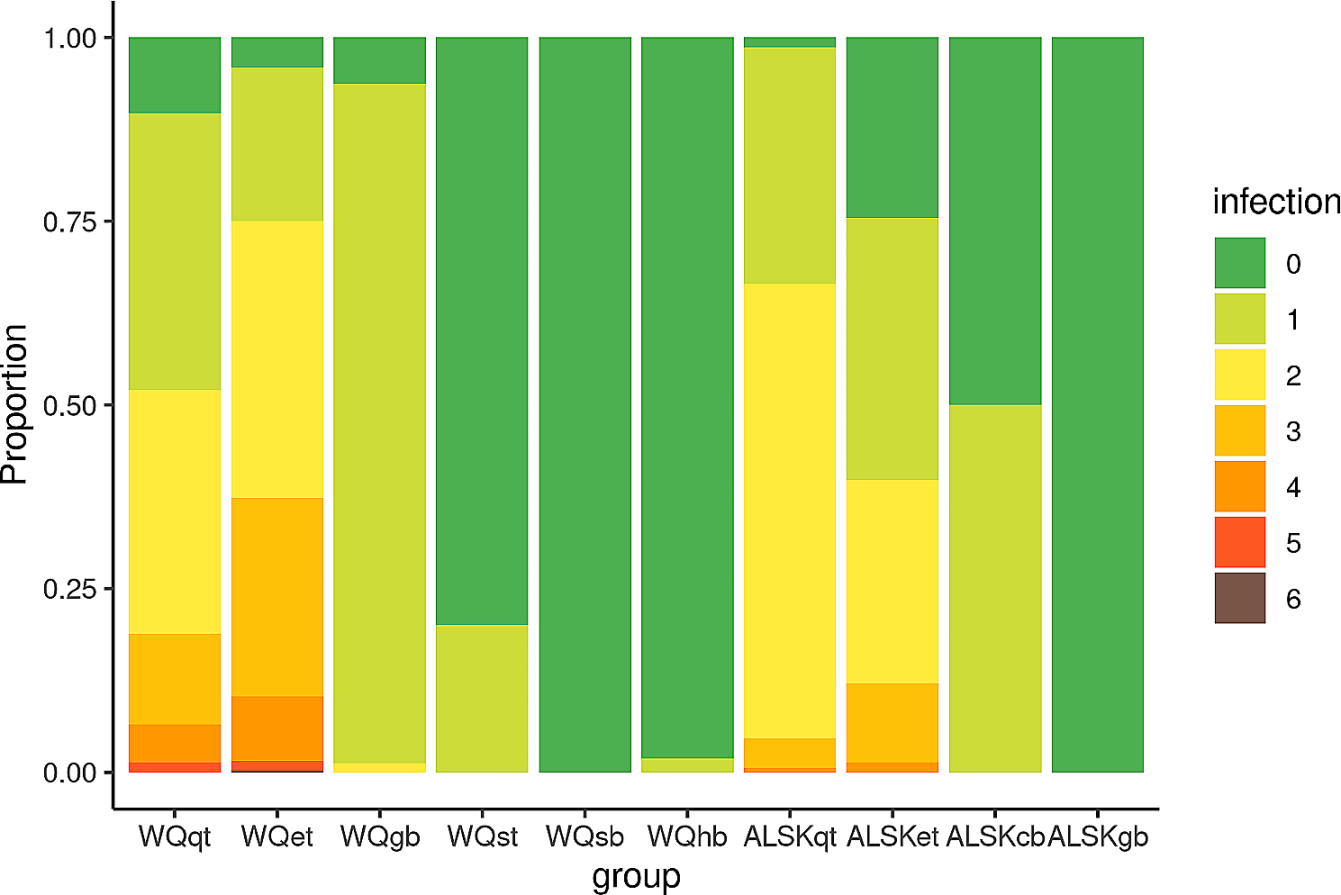
The proportion of the different coexist number of microorganisms in different sample types. Different color column represents the different coexist number of microorganisms in that sample type. WQqt: questing ticks from WQ; WQet: engorged ticks from WQ; WQgb: goat blood from WQ; WQst: squirrel’s ticks from WQ; WQsb: squirrel blood from WQ; WQhb: human blood from WQ; ALSKqt: questing ticks from ALSK; ALSKet: engorged ticks from ALSK; ALSKcb: cattle blood from ALSK; ALSKgb: gerbil blood from ALSK

A total of 1226 *Coxiella spp.* infection cases were detected, and all these cases were ticks collected from the two counties. All *Coxiella spp.* had the same sequence, which was homologous to the sequence of uncultured *Coxiella sp.* clone Dx-56 and that of JX432012.1, indicating that they were tick *Coxiella*-like symbionts. The carrying rates of *Coxiella spp.* in ticks collected were 80.83% (485/600) for WQqt, 76.75% (393/512) for WQet from goats, 10% (2/20) for WQrt, 42.91% (245/570) for ALSKet from livestock cattle, and 66.45% (101/152) for ALSKqt. These results suggest that the *Coxiella*-like symbionts are widely distributed and stable in the tick populations in the two regions. According to the number of detected microorganisms, most of the pathogen-carrying blood samples only carried one detected microorganism, and only one case was detected in the Wenquan goat blood sample carrying both *Ehrilichia spp.* and *Anaplasma ovis*. However, tick samples often carry up to 6 pathogens (Fig. 6).

## Discussion

The microbiome of a variety of ticks has been described and characterized in previous reports [53, 54], and some of the components, such as *Francisella/Coxiella* symbionts, have been shown to be physiologically significant for ticks [10]. This study showed that the natural patterns of tick microbial communities is dynamic and unstable, the living environment and parasitic state have a significant impact on the microbiota composition and bacterial cooccurrence pattern of ticks. The microbial cooccurrence network takes environmental microorganisms as the central node, and no consistency is found between different groups(Special attention: In order to minimize the possibility of detecting contamination, tick samples are carefully cleaned with 70% ethanol and phosphate-buffered saline (PBS PH7.4) after collection to prevent any potential contamination of DNA by environmental microorganisms.). In addition, and explored a possible approach to control ticks or pathogens by manipulating the tick microbial community.

### Metabolic pathways of tick-carrying bacteria

There is an intimate relationship between insect vectors and the microorganisms they carry [55]. The symbiotic bacteria of ticks have been shown to provide chemical molecules, such as chorismite [56]\5-HT [57]\B vitamins [9]\cbb3-cyt b oxidase [58]\ATP [58], to help ticks develop. In this study, the results indicated that the microorganisms carried by different tick species collected from same area has no preference. There were nine pathways involved in the parasitic state change, and three of them, the chorismate biosynthesis I, chorismate biosynthesis from 3-dehydroquinic acid, and aromatic amino acid synthesis pathways, have been shown to be associated with promoting blood meal appetite [57]. The results also suggest that changes in parasitic status did not just cause appetite changes. However, the biological significance of the pathway changes needs further study.

### No stable pattern indicated by co-occurrence networks

Co-occurrence networks are widely used to study relationships between microbial communities [59]. Our results showed that the co-occurrence network of tick-carrying microorganisms were significantly affected by the grow location of ticks, their blood saturation level, and developmental status, which indicates that the tick-carrying microorganisms could not form a stable community in the host. Studies of laboratory-raised ticks have directly shown that ticks can acquire microorganisms from the environment and blood meal [8]. It can be reasoned that the acquisition of environmental microorganisms and the changes in the physiological environment of ticks before and after their blood meal could be the main reasons for the changes in the diversity and abundance of tick-borne microorganisms. In addition, the existence of mutualism, symbiosis, competition, and antagonism among microorganisms may result the differences in microbial composition in the ticks.

### *Coxiella* symbionts

It has been reported that *Coxiella* symbionts are involved in the life activities of ticks in many ways [57, 59]. We found that the blood saturation level of ticks significantly affected the abundance of *Coxiella* symbionts and the abundances of 3 pathways related to blood meal behavior. We also found that *Coxiella* symbionts were associated with 10 pathogens. These results suggest that ticks carrying a high abundance of *Coxiella* may carry a higher abundance of related pathogens, although we don’t know if *Coxiella* influences pathogens, or vice versa. But a possible point is that inhibiting *Coxiella* abundance may control the tick borne pathogens. On the one hand, ticks with low *Coxiella* abundance have lower willingness to find hosts for blood meal. On the other hand, low *Coxiella* abundance may be not sufficient for related pathogen enrichment. Interestingly, *Phenylobacterium* was the negatively correlated bacterium with the *Coxiella* abundance. It has been reported that *Phenylobacterium* can be isolated from environmental samples and can only be enriched in media containing benzene ring drugs [60], which means that an environment with a high abundance of *Phenylobacterium* may not be beneficial to the enrichment of tick-carrying *Coxiella*. Thus, high concentrations of benzene-containing compounds may be able to control the abundance of tick-carrying *Coxiella* to reduce the blood-sucking appetite of ticks and control tick population.

### Carrying and infection of different tick-borne pathogens

The process by which ticks transmit pathogens is affected by many factors [61, 62]. A high tick-borne pathogen carrying rate increases the probability of transmitting pathogens by ticks, but it will be affected by many factors, such as the number of pathogens carried, the suitability of hosts to the pathogens, the immune clearance ability of the hosts, and the immune evasion ability of the pathogens. Our research showed that *Rickettsia* had a low infection rate in animals and humans, which may be related to the content of the pathogen and the immunity of the host to the pathogen [63, 64]. In addition, *Anaplasma ovis* had a high infection rate in suitable host goat while the *Anaplasma phagocytophilum* could not be found in hosts. Thus, the suitable host, high tick carrying rate and high pathogen abundance cause a large-scale infection of *Anaplasma ovis* in goat while the zero infections occurring in non-suitable hosts could be related to immunity [65]. The infection rate of *Babesia* in livestock is higher than that in ticks in the Alataw City area, which may be due to the long existence of *Babesia* in host [66]. Moreover, *F. tularensis* is a highly virulent zoonotic pathogens [67], and inhalation of 10 CFU of *F. tularensis* can cause severe disease. Therefore, prevention and control of *Francisella tularensis subsp* transmission are still critical.

### Limitation

Although we found a negative or positive correlation between the abundance of the *Coxiella* symbiont and some environmental bacteria or related pathogens by the abundance linear regression model, this finding needs to be further confirmed by more tests in the future.

## Conclusions

This study shows that the species and quantities of bacteria carried by ticks are diverse as they are affected by the environment and parasitic states. The intracellular symbiotic bacteria, *Coxiella/Francisella*, shows the most variations in the abundance of the species and quantities. Meanwhile, *Coxiella* abundance is linearly correlated with the abundance of its associated microbes. Moreover, *Phenylobacterium* had a strong negative correlation with the symbiont *Coxiella*. In addition, tick-borne microbes endow ticks with several metabolic functions and the abundance of metabolic pathways of bacteria is affected by the environment and the ticks’ blood saturation levels. Thus, no stable coexistence relationship was determined in the tick-carrying microbiota coexistence network. The carrying rate of tick-borne pathogens was very high, and there were significant differences among different tick borne pathogens. Taken together, the microbes in the environment and the egg-borne symbionts play an important role in the composition of tick microbial communities. This study provides directions for further understanding and utilization of tick microbiomes, as well as control strategies for ticks and tick-borne pathogens.

### Electronic supplementary material

Below is the link to the electronic supplementary material.

Supplementary Material 1

Supplementary Material 2

Supplementary Material 3

Supplementary Material 4

Supplementary Material 5

Supplementary Material 6

Supplementary Material 7

Supplementary Material 8

Supplementary Material 9

Supplementary Material 10

Supplementary Material 11

Supplementary Material 12

Supplementary Material 13

Supplementary Material 14

## Data Availability

Sequence data that support the findings of this study have been deposited to to SRA with the BioProject ID PRJNA1048745(https://www.ncbi.nlm.nih.gov/bioproject/PRJNA1048745). The datasets supporting the conclusions of this article are included within the article and its additional files.

## Abbreviations

WQ: Wenquan county
ALSK: Alataw City
NMDS: Nonmetric multidimensional scaling
ANOVA: Analysis of variance
ANOSIM: Analysis of similarities
LEfSe: Linear discriminant analysis effect size
ASV: Amplicon sequence variant
CoNets: Co-occurrence networks
SparCC: Sparse Correlations for Compositional data
MLRM: Multiple linear regression model
