# Supplementary material for: The bacterial patterns suggesting the dynamic features of tick-associated microorganisms in hard ticks
Source: BMC Microbiol. 2024 May 24;24:179. doi: 10.1186/s12866-024-03323-3 (PMC11118998; doi:10.1186/s12866-024-03323-3)
Supplement: Supplementary file 5 — Supplementary Material 5 [file 12866_2024_3323_MOESM5_ESM.pdf]

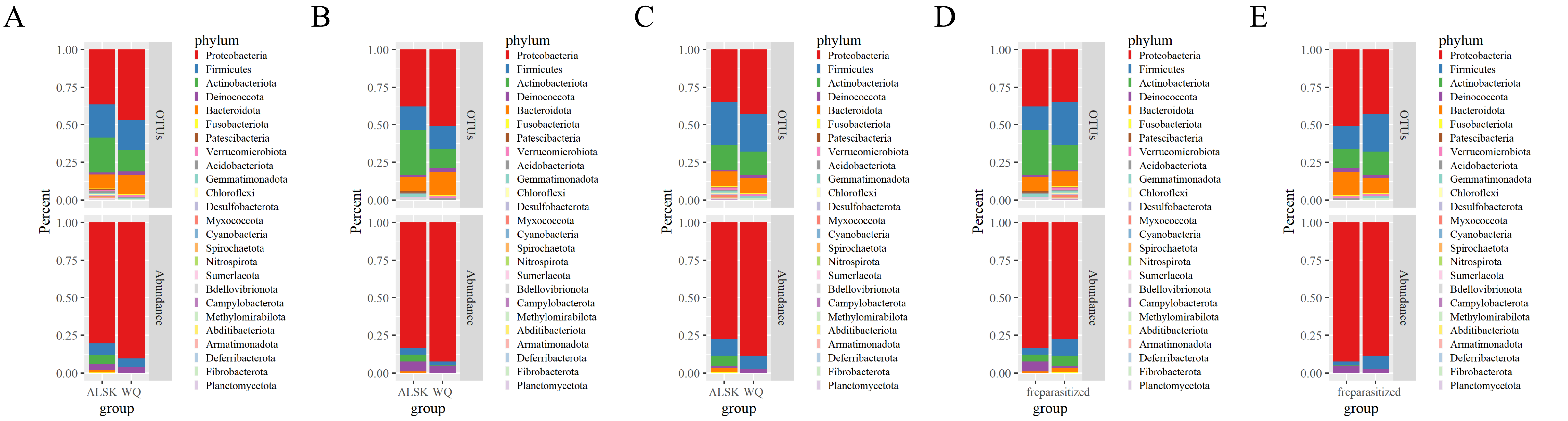

A

phylum	W	*P* value	comparison	sig.
Abditibacteriota	1540	NaN	<	
Acidobacteriota	1681.5	0.048404	>	*
Actinobacteriota	2875	3.35E-15	>	*
Armatimonadota	1540	NaN	<	
Bacteroidota	2730.5	1.62E-12	>	*
Bdellovibrionota	1624	0.079578	>	
Campylobacterota	1568	0.32167	>	
Chloroflexi	1568	0.32167	>	
Cyanobacteria	1652	0.041687	>	*
Deferribacterota	1540	NaN	<	
Deinococcota	842	3.89E-05	<	*
Desulfobacterota	1652	0.041687	>	*
Fibrobacterota	1512.5	0.330547	<	
Firmicutes	2025	0.004269	>	*
Fusobacteriota	1796	0.006963	>	*
Gemmatimonadota	1711	0.042891	>	*
Methylomirabilota	1568	0.32167	>	
Myxococcota	1624	0.079578	>	
Nitrospirota	1568	0.32167	>	
Patescibacteria	2212	3.80E-08	>	*
Planctomycetota	1540	NaN	<	
Proteobacteria	916	0.000236	<	*
Spirochaetota	1652	0.041687	>	*
Sumerlaeota	1736	0.006205	>	*
Verrucomicrobiota	1746	0.019404	>	*

B

phylum	W	*P* value	comparison	sig.
Abditibacteriota	262.5	NaN	<	
Acidobacteriota	325	0.011701	>	*
Actinobacteriota	447	9.00E-05	>	*
Armatimonadota	262.5	NaN	<	
Bacteroidota	426.5	0.000511	>	*
Bdellovibrionota	280	0.13784	>	
Campylobacterota	262.5	NaN	<	
Chloroflexi	262.5	NaN	<	
Cyanobacteria	297.5	0.031476	>	*
Deferribacterota	262.5	NaN	<	
Deinococcota	274	0.817885	>	
Desulfobacterota	262.5	NaN	<	
Fibrobacterota	262.5	NaN	<	
Firmicutes	347	0.075168	>	
Fusobacteriota	255	0.541193	<	
Gemmatimonadota	297.5	0.031476	>	*
Methylomirabilota	262.5	NaN	<	
Myxococcota	262.5	NaN	<	
Nitrospirota	262.5	NaN	<	
Patescibacteria	420	6.99E-07	>	*
Planctomycetota	262.5	NaN	<	
Proteobacteria	179	0.078793	<	
Spirochaetota	262.5	NaN	<	
Sumerlaeota	280	0.13784	>	
Verrucomicrobiota	255	0.541193	<	

C

phylum	W	*P* value	comparison	sig.
Abditibacteriota	420	NaN	<	
Acidobacteriota	441	0.313167	>	
Actinobacteriota	807.5	4.24E-09	>	*
Armatimonadota	420	NaN	<	
Bacteroidota	775	5.63E-08	>	*
Bdellovibrionota	441	0.313167	>	
Campylobacterota	430.5	0.490153	>	
Chloroflexi	430.5	0.490153	>	
Cyanobacteria	441	0.313167	>	
Deferribacterota	420	NaN	<	
Deinococcota	188	0.000439	<	*
Desulfobacterota	462	0.142096	>	
Fibrobacterota	400	0.178423	<	
Firmicutes	474	0.419853	>	
Fusobacteriota	516	0.036748	>	*
Gemmatimonadota	443	0.560443	>	
Methylomirabilota	430.5	0.490153	>	
Myxococcota	451.5	0.209221	>	
Nitrospirota	430.5	0.490153	>	
Patescibacteria	577.5	0.001615	>	*
Planctomycetota	420	NaN	<	
Proteobacteria	269	0.021465	<	*
Spirochaetota	462	0.142096	>	
Sumerlaeota	483	0.066381	>	
Verrucomicrobiota	499	0.064567	>	

D

phylum	W	*P* value	comparison	sig.
Abditibacteriota	300	NaN	<	
Acidobacteriota	363	0.029085	>	*
Actinobacteriota	246.5	0.316525	<	
Armatimonadota	300	NaN	<	
Bacteroidota	211	0.094403	<	
Bdellovibrionota	306	0.791712	>	
Campylobacterota	292.5	0.567628	<	
Chloroflexi	292.5	0.567628	<	
Cyanobacteria	327	0.266038	>	
Deferribacterota	300	NaN	<	
Deinococcota	493	0.000272	>	*
Desulfobacterota	270	0.215662	<	
Fibrobacterota	300	NaN	<	
Firmicutes	229	0.1848	<	
Fusobacteriota	217.5	0.026548	<	*
Gemmatimonadota	299	0.987706	>	
Methylomirabilota	292.5	0.567628	<	
Myxococcota	277.5	0.290807	<	
Nitrospirota	292.5	0.567628	<	
Patescibacteria	373	0.130518	>	
Planctomycetota	300	NaN	<	
Proteobacteria	357	0.289021	>	
Spirochaetota	270	0.215662	<	
Sumerlaeota	272	0.369488	<	
Verrucomicrobiota	232.5	0.049369	<	*

E

phylum	W	*P* value	comparison	sig.
Abditibacteriota	367.5	NaN	<	
Acidobacteriota	378	0.46069	>	
Actinobacteriota	480	0.057082	>	
Armatimonadota	367.5	NaN	<	
Bacteroidota	525	0.005886	<	*
Bdellovibrionota	367.5	NaN	<	
Campylobacterota	367.5	NaN	<	
Chloroflexi	367.5	NaN	<	
Cyanobacteria	367.5	NaN	<	
Deferribacterota	367.5	NaN	<	
Deinococcota	525	0.007112	>	*
Desulfobacterota	367.5	NaN	<	
Fibrobacterota	350	0.209802	<	
Firmicutes	209	0.006738	<	*
Fusobacteriota	360	0.712536	<	
Gemmatimonadota	332.5	0.069379	<	
Methylomirabilota	367.5	NaN	<	
Myxococcota	367.5	NaN	<	
Nitrospirota	367.5	NaN	<	
Patescibacteria	367.5	NaN	<	
Planctomycetota	367.5	NaN	<	
Proteobacteria	413	0.449568	>	
Spirochaetota	367.5	NaN	<	
Sumerlaeota	367.5	NaN	<	
Verrucomicrobiota	360	0.712536	<	

OTU composition and abundance comparisons at phylum level. OTU composition and abundance comparisons for **A**, tick-carrying bacteria from ALSK and WQ; **B**, tick-carrying bacteria of free ticks from ALSK and WQ; **C**, tick-carrying bacteria of engorged ticks from ALSK and WQ; **D**, tick-carrying bacteria of free and engorged ticks from ALSK; **E**, tick-carrying bacteria of free and engorged ticks from WQ. Tables below stack plots are statistical parameters.
