# Supplementary material for: The bacterial patterns suggesting the dynamic features of tick-associated microorganisms in hard ticks
Source: BMC Microbiol. 2024 May 24;24:179. doi: 10.1186/s12866-024-03323-3 (PMC11118998; doi:10.1186/s12866-024-03323-3)
Supplement: Supplementary file 11 — Supplementary Material 11 [file 12866_2024_3323_MOESM11_ESM.pdf]

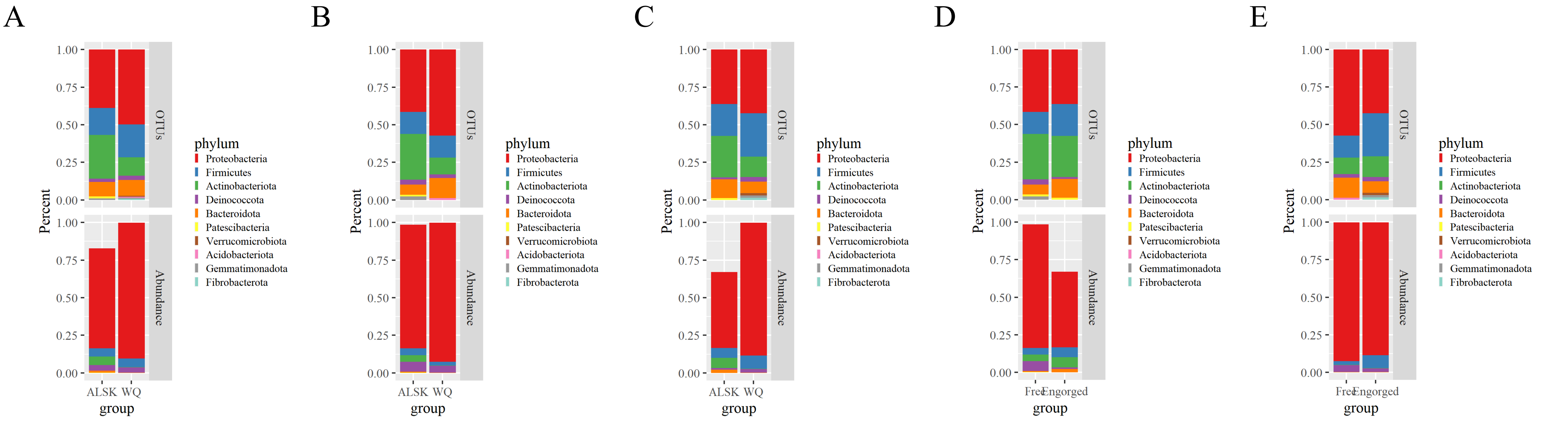

A

phylum	W	*P* value	comparison	sig.
Acidobacteriota	1458	0.330793	<	
Actinobacteriota	2777	4.73E-15	>	*
Bacteroidota	2613	5.75E-12	>	*
Deinococcota	800	3.34E-05	<	*
Fibrobacterota	1458	0.330793	<	
Firmicutes	1549	0.700347	>	
Gemmatimonadota	1459.5	0.592917	>	
Patescibacteria	2145	3.55E-08	>	*
Proteobacteria	461	5.54E-10	<	*
Verrucomicrobiota	1458	0.330793	<	

B

phylum	W	*P* value	comparison	sig.
Acidobacteriota	231	0.551273	<	
Actinobacteriota	417	4.64E-05	>	*
Bacteroidota	393	0.000423	>	*
Deinococcota	262	0.598079	>	
Fibrobacterota	238	NaN	<	
Firmicutes	329	0.039066	>	*
Gemmatimonadota	255	0.130393	>	
Patescibacteria	391	3.78E-07	>	*
Proteobacteria	134	0.017615	<	*
Verrucomicrobiota	238	NaN	<	

C

phylum	W	*P* value	comparison	sig.
Acidobacteriota	390	NaN	<	
Actinobacteriota	749.5	8.99E-09	>	*
Bacteroidota	709	2.71E-07	>	*
Deinococcota	172	0.000488	<	*
Fibrobacterota	370.5	0.173634	<	
Firmicutes	289	0.107533	<	
Gemmatimonadota	351	0.049269	<	*
Patescibacteria	540	0.001754	>	*
Proteobacteria	112	2.22E-06	<	*
Verrucomicrobiota	370.5	0.173634	<	

D

phylum	W	*P* value	comparison	sig.
Acidobacteriota	273	NaN	<	
Actinobacteriota	236.5	0.467662	<	
Bacteroidota	219	0.280405	<	
Deinococcota	458	0.000193	>	*
Fibrobacterota	273	NaN	<	
Firmicutes	315	0.402425	<	
Gemmatimonadota	292.5	0.103897	>	
Patescibacteria	356	0.068762	>	
Proteobacteria	418	0.00281	>	*
Verrucomicrobiota	273	NaN	<	

E

phylum	W	*P* value	comparison	sig.
Acidobacteriota	350	0.466236	>	
Actinobacteriota	440	0.073485	>	
Bacteroidota	463	0.022363	<	*
Deinococcota	487	0.007855	>	*
Fibrobacterota	323	0.205695	<	
Firmicutes	188	0.005896	<	*
Gemmatimonadota	306	0.066676	<	
Patescibacteria	340	NaN	<	
Proteobacteria	390	0.378543	>	
Verrucomicrobiota	323	0.205695	<	

OTU composition and abundance comparisons of CoNets at phylum level. OTU composition and abundance comparisons for **A**, tick-carrying bacteria from ALSK and WQ; **B**, tick-carrying bacteria of free ticks from ALSK and WQ; **C**, tick-carrying bacteria of engorged ticks from ALSK and WQ; **D**, tick-carrying bacteria of free and engorged ticks from ALSK; **E**, tick-carrying bacteria of free and engorged ticks from WQ. Tables below stack plots are statistical parameters.
